# Mr. Simmons, on Vaccine

**Published:** 1801-02

**Authors:** W. Simmons

**Affiliations:** Manchester


GENTLEMEN,

I Obſerve that diſcuſſions on the Cow-pox ſtill continue to occupy ſeveral pages in each Number of your Journal. Perhaps no ſubject ever met with ſo ample an examination within ſo ſhort a time; and indeed its importance to the welfare of mankind is commenſurate to the pains beſtowed upon it. The real merits of the diſcovery, however, ſeem to lie within a narrow compaſs, and to reſt chiefly on two points: Firſt, whether it be a ſecurity againſt the Small-pox? and, ſecondly, whether it be a milder diſeaſe? If the teſtimony of medical men in its favour, more general than ever was before publiſhed on any one profeſſional ſubject, can be ſuppoſed to determine the former, it muſt be admitted as proved beyond all controverſy; and, with regard to the latter, the reſult of unambiguous experience is alike convincing. Theſe two points then being as ſatisfactorily proved as any others in medicine, it is unfair to charge the diſcovery itſelf with miſtakes which have occurred in conducting the inoculation.

The ſources of error in inoculating for the Cow-pox, appear to have ariſen from miſtaking the proper time for taking the fluid, and by not noting the characteriſtic progreſs of the diſeaſe on the part inoculated. In my own practice I have obſerved, that the veſicle contained the largeſt quantity of fluid on the tenth day, including that of inoculation, and, on the eleventh, that it had nearly diſappeared, and what little remained had become inert; at leaſt, when taken in that ſtage, it has failed with me in exciting the diſeaſe, in the ſeveral inſtances in which I have tried it. There is ſome variety however in the courſe of the diſeaſe, and the ninth day may, in ſome caſes, be more proper for taking it, as, in others, I have found the eleventh and twelfth to be. About a month ſince I inoculated a boy for the Cow-pox, and till the tenth day I was not certain that he had taken the infection; the changes on the arm were thence very rapid, and no fluid could be diſcerned in the veſicle on the thirteenth. His two brothers, both younger than himſelf, were inoculated from the ſame thread, at the ſame time, and went through the diſeaſe in the uſual way.

Much apprehenſion has been entertained of an extenſive inflammation ſpreading from the inoculated part, and of a conſequent ulceration. In my own practice, the inflammation has ſometimes been conſiderable, but it has not furniſhed me with a ſingle a fingle inftance of a troublefome degree of ulceration. In feveral, where the inflammatory appearances have been threatening, I have thought that they have been aggravated by the tightnefs of the drefs, and accordingly on ordering a loofe veft, they have difappeared in due time. But in the worft cafes I have yet feen, no other application has been neceffary than the vegeto-mineral water.

An eruption of puftules was, for fome time, a pretty conftant attendant on this inoculation; latterly, however, feldom any have appeared on diftant parts of the body, and not more in number than three or four; the inflammation on the arm, together with a puftular eruption on that part, and a flight febrile attack, have conftituted all the figns of difeafe. The true character of the Cow-pox had undoubtedly been changed by an intermixture of the variolous and vaccine virus.

Frequent difappointment has arifen from the fluid employed for inoculation having loft its activity; this may be obviated in future, by inoculating immediately from the ripe veficle; or, where that cannot be complied with, the chance of mifcarriage may be leffened by faturating a thread, and keeping it in a well-corked vial till the time of ufing; for, when taken upon a lancet it foon becomes inert. There is a ftriking difference in this refpect, between variolous matter and the vaccine; which I have been accuftomed to explain by fuppofing that, in the Small-pox, the active particles are fo enveloped in pus, as to prevent their fudden exhalation or decompofition; whereas, in the Cow-pox, no fuppuration takes place, and the fluid being but little more vifcid than water, is foon deprived of its peculiar properties by the fpecific portion flying off, or by undergoing fome chemical alteration.

Though no inftance of a violent or unmanageable degree of inflammation or ulceration has occurred to me, the experience of others proves, that both are fometimes very troublefome and alarming. As I cannot write upon this point from my own obfervation, I fhould have ftopped here; but, reafoning from analogy, I am led to fufpect, that in thofe cafes where either one or other of the above fymptoms, particularly the former, has taken place in a violent degree, the future fecurity of the patient againft the variolous contagion muft be rendered fomewhat doubtful. The inflammation on the part, and fymptoms of general irritation defcribed by writers, refemble thofe produced by the abforption of putrid matter from a wound, or what are caufed by a fimple incifion in a bad habit of body, and are effentially different from the regular courfe of the difeafe. Thefe bear a clofe analogy to a chancre happening in a bad conftitution, and followed by inflammation terminating in gangrene, or in an ulcer difficult of cure. How violent foever thefe fymptoms may be, the fyftem not uncommonly efcapes contamination by the venereal poifon; and therefore I hold it fafe, when the inflammatory appearances on the arm greatly exceed the ufual limits, that the Vaccine Inoculation fhould be repeated at a convenient diftance of time, to guard againft all chance of difappointment. It is only after the changes upon the inoculated arm have been regular and well defined, that a patient can be faid to have fecurely undergone the difeafe.

On the origin of the Cow-pox, conjecture has been bufily employed; but, whether it originate in the horfe or in the cow, or by the united influence of both, does not appear to be of much practical importance. The objections derived from the loathfomenefs of the origin of it in the horfe, would, by parity of reafoning, reject the ufe of pork and of duck for food, becaufe fwine and ducks are foul feeders. It is not the fource whence it may be derived, fo much as the ufeful purpofes to which it may be applied in its mature ftate that claim our attention. We fhould be little folicitous whether mercury were derived from the animal or vegetable inftead of the mineral kingdom, whilft we know that it is an antidote to the venereal poifon. However, as an object of curiofity, and as opening a new field for phyfiological inquiry, the fuggeftion may be fubmitted again to the teft of experiment, the only mode of afcertaining its truth or fallacy. In thofe experiments which I publifhed on this fubject two years ago, every attention of which I am capable was paid to accuracy, and the refult was decidedly againft that opinion. Lately, the matter of greafe has been faid to have been employed with fuccefs; but furely where the genuine Cow-pox fluid, of any age, had been fuffered to come in contact with the ulcerated furface, it muft throw an ambiguity over the whole. An experiment fo loofely conducted, may deferve repetition, from the reported contradicton of former facts, and the feeming fingularity of the refult; but cannot be deemed conclufive againft the experiments of others, as well as myfelf to the contrary. Neverthelefs, I am not at all tenacious of my opinion, and fhall readily relinquifh it, on feeing proper grounds for a conviction of its error.

Befides the two cardinal points of fecurity and mildnefs, the Cow-pox poffeffes other properties of a very ufeful though fubordinate nature; it is not contagious; it neither ftirs up fcrofula, nor any other difpofition to difeafe that may lie dormant in the fyftem; nor with itfelf is any other introduced; and it may be communicated at all ages, and under all circumftances in which a rational practitioner would think of communicating it. By the general introduction of this practice, the comfortable hope of exterminating the Small-pox may be reafonably entertained, and thus millions of lives yet unborn be preferved to their friends and fociety.

We learn by the accounts from the Mediterranean, that the Cow-pox preferves its characteriftic mildnefs even in a more fouthern latitude; where, when it appears, the Small-pox is dreadfully deftructive. This is particularly the cafe upon the coaft of Africa, and on board our flave-fhips; to obviate which in future, I would recommend the introduction of the Cow-pox, and that every flave fhould be inoculated for it, as foon as taken on board. By this means their lives would be preferved, and their value enhanced by the fecurity, when arrived at the place of their deftination. The moft beneficial confequences might be expected alfo from introducing it among the flaves in the Weft Indies, as the Small-pox is likewife very deftructive to them in that climate.

I have put together thefe few obfervations, in order to add my teftimony in detail, in favour of the new practice, to the mafs of evidence already before the public; and to pay my tribute of refpect thus publicly to Dr. Jenner for the original fuggeftion, and to Dr. Pearfon and others for the zeal and induftry with which they have promoted the practice of this valuable difcovery. Should they be deemed of fufficient importance to merit a place in your Journal, they are entirely at your difpofal. I am, &c.

W. SIMMONS.

*Manchefter,*

*December* 9, 1800.

---

